# Noninvasive malignant mammary adenomyoepithelioma in a male cat

**DOI:** 10.1007/s11259-026-11493-w

**Published:** 2026-09-04

**Authors:** Felipe Martins Pastor, Enio Ferreira, Daiana Yively Osório Taborda, Nayara Toledo da Silva Martinez, Geovanni Dantas Cassali, Rogéria Serakides, Natália Melo Ocarino

**Affiliations:** 1 Departamento de Clínica e Cirurgia Veterinárias, Escola de Veterinária, Universidade Federal de Minas Gerais, Avenida Antônio Carlos 6627, 31270-901 Belo Horizonte, Minas Gerais Brasil; 2 Departamento de Patologia Geral, Instituto de Ciências Biológicas, Universidade Federal de Minas Gerais, Avenida Antônio Carlos 6627, 31270-901 Belo Horizonte, Minas Gerais Brasil

**Keywords:** Mammary tumors, Feline, Immunohistochemistry, CK, P63, Cox-2

## Abstract

This report describes the histopathological and immunohistochemical findings of a noninvasive malignant mammary adenomyoepithelioma in a seven-year-old neutered male mixed-breed cat. The animal was euthanized due to an extensive mandibular fracture caused by squamous cell carcinoma. Macroscopically, a 3 × 1.5 cm, elevated, well-defined, ulcerated, lobulated, reddish-white, solid – occasionally cystic – and firm nodule was observed in the right inguinal mammary gland. Microscopically, an expansive neoplastic proliferation composed of myoepithelial and epithelial cells arranged predominantly in a papillary pattern was observed. Immunohistochemistry was performed for the markers C-Kit, pan-cytokeratin (CK-AE1/AE3), cytokeratin 7 (CK7), P63, estrogen receptor (ER), progesterone receptor (PR), Ki67, cyclooxygenase-2 (Cox-2), and GATA3. The neoplastic cells were positive for CK AE1/AE3 and 7 (epithelial cells), P63 (myoepithelial cells), Cox-2 (epithelial and myoepithelial cells), and PR (epithelial cells). Positive results for CK-AE1/AE3, CK7, and P63 confirmed the epithelial and myoepithelial nature of the tumor, the mitotic index and elevated Ki-67 expression supported the interpretation of a malignant proliferative phenotype and contributed to the classification of the lesion as a noninvasive malignant adenomyoepithelioma. Based on the macroscopic, microscopic, and immunohistochemical findings, a diagnosis of noninvasive malignant mammary adenomyoepithelioma was established.

## Introduction

Mammary tumors are the third most common neoplasms in felines, following skin and hematopoietic tumors (Cassali et al. 2024; Goldschmidt et al. 2016). In female cats, mammary neoplasms occur between 10 and 11 years of age (Goldschmidt et al. 2016), and 80%–90% of tumors are malignant, with carcinoma being the most frequent (Nunes et al. 2021). In humans and domestic animals, mammary tumors are uncommon in males. Due to their low incidence, few reports are available on these diseases in dogs and cats (Rodrigues et al. 2023). In male cats, available reports include descriptions of non-neoplastic lesions, such as fibroepithelial hyperplasia (Bonatto et al. 2021; Küçükbekir et al. 2020; Mayayo et al. 2018; Rodrigues et al. 2023; Voorwald et al. 2021), and neoplasms, such as adenoma and carcinomas (Jacobs et al. 2010; Pires et al. 2008; Pîrvu et al. 2024; Skorupski et al. 2005; Spugnini et al. 2010).

Adenomyoepithelioma is a mammary tumor microscopically characterized by neoplastic proliferation of epithelial and myoepithelial cells. In most cases, these tumors are benign; however, although rare, malignant transformations can occur in one or both cellular components in humans and animals (Howlett et al. 2003; Jones et al. 2003; Nunes et al. 2021). The growth pattern of this tumor can be heterogeneous because of the variable proportions of epithelial and myoepithelial cells that form nests, tubules, trabeculae, and/or papillary projections (Goldschmidt et al. 2011; Jones et al. 2003; Rakha et al. 2021). In cats, malignant adenomyoepithelioma has only been reported in female cats (Campos et al. 2015; Nunes et al. 2021). To the best of our knowledge, there are no previous reports of malignant mammary adenomyoepithelioma in male cats, making this the first such case described in the literature.

This study aimed to describe the histopathological and immunohistochemical findings in a male cat with noninvasive malignant mammary adenomyoepithelioma.

## Case report

A neutered and mixed-breed seven-year-old male cat was euthanized because of a poor prognosis resulting from an extensive fracture in the left jaw associated with oral cavity neoplasia. Necropsy revealed a closed, complete, and comminuted fracture of the left mandible in the region of the premolar teeth with hemorrhage in the adjacent soft tissues. As confirmed by histopathological analyses, the fracture was due to tumor invasion and bone necrosis caused by a squamous cell carcinoma of the oral cavity.

**Fig. 1:**
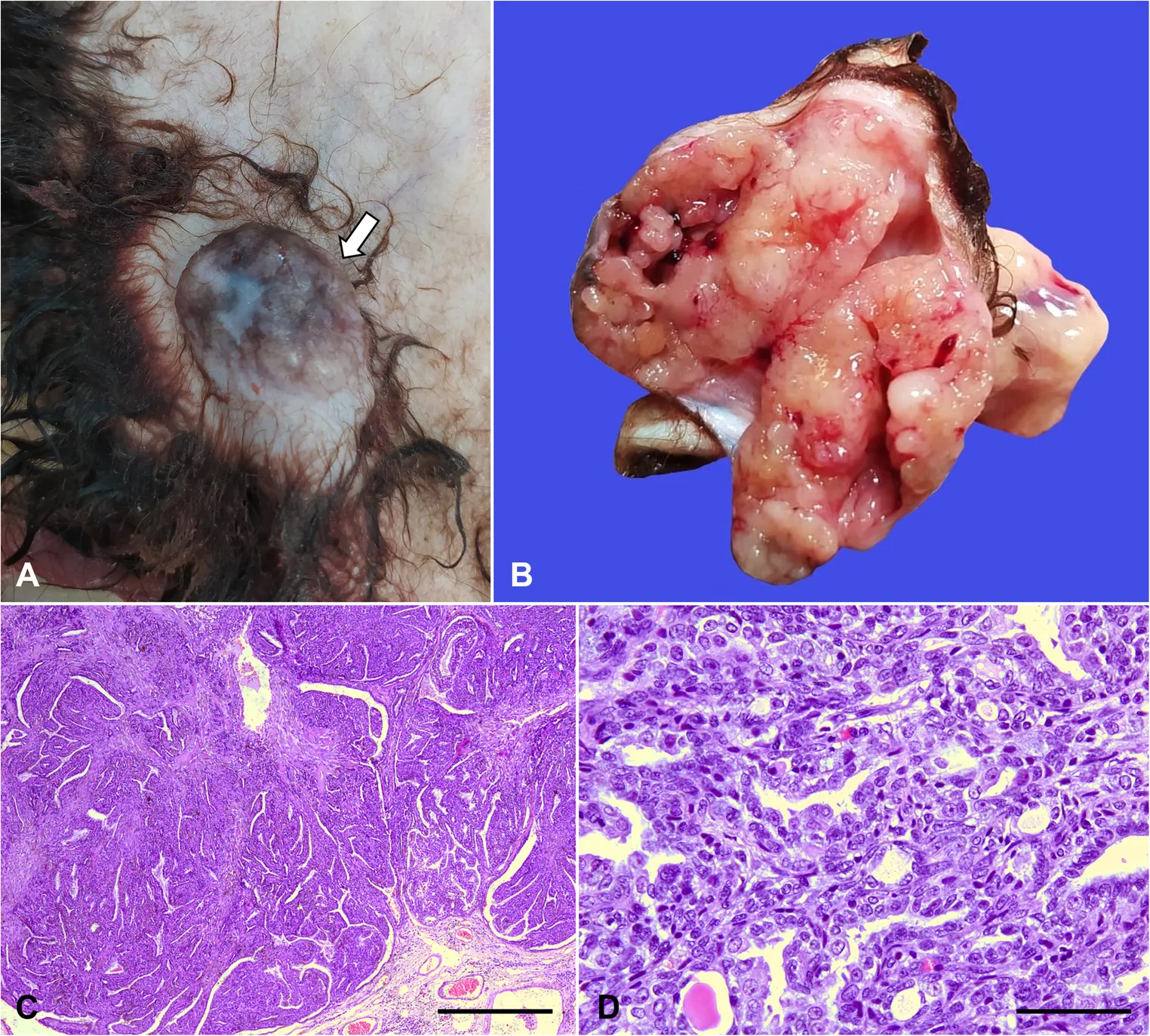
Macroscopic and microscopic appearance, noninvasive malignant mammary adenomyoepithelioma in a male cat. (**A**) Right inguinal mammary region, a nodule measuring approximately 3 × 1.5 cm in length, elevated, well-defined, and firm (arrow). (**B**) Cut surface, nodule with lobulated appearance, reddish white, and sometimes cystic. (**C**) Microscopic appearance, nodule consisted of a well-defined, encapsulated, expansive, and densely cellular proliferation of neoplastic epithelial and myoepithelial cells. HE, bar: 500 μm. (**D**) Epithelial cells arranged in the tubules and papillae, with a single or double layer of round to cuboidal cells surrounded by bundles of myoepithelial cells. HE, bar: 50 μm

In the right inguinal mammary region, a nodule measuring approximately 3 × 1.5 cm in length, elevated, well-defined, ulcerated and firm was observed; when cut, it had a lobulated appearance, reddish white in color, and sometimes cystic (Fig. 1A, B).

Fragments of nodules and thoracic and abdominal organs were collected in a 10% buffered formalin solution, processed using a routine paraffin embedding technique, and stained with hematoxylin-eosin for histopathological evaluation.

The nodule consisted of a well-defined, encapsulated, expansive, and densely cellular proliferation of neoplastic epithelial and myoepithelial cells originated in the mammary gland tissue (Fig. 1C). Epithelial cells were arranged in the tubules and papillae, with a single or double layer of round to cuboidal cells surrounded by bundles of myoepithelial cells (Fig. 1D). The cytoplasm was poorly delimited, slightly eosinophilic, and homogeneous, with a round or oval central or paracentral nucleus, finely dotted chromatin, and a single central nucleolus. Moderate cellular and nuclear pleomorphism were observed, and eight mitotic figures in 10 high power fields (HPF) (40×, 2.37mm^2^). The myoepithelial population was arranged in bundles, surrounded by a moderate amount of weakly eosinophilic extracellular matrix. The cells were elongated and large, with partially visible cytoplasmic boundaries. The cytoplasm was scant, eosinophilic, and sometimes vacuolated, whereas the nucleus was oval, elongated, and large, with dotted chromatin and visible nucleoli. Moderate cellular and nuclear pleomorphism, and six mitotic figures in 10 HPF (40×, 2.37mm^2^) were observed. No necrotic or vascular invasive areas were observed. No metastasis was observed.

For a definitive diagnosis, histological sections were evaluated using an immunohistochemical panel (Table 1). For immunohistochemistry, 3 μm-thick tissue sections were prepared on silanized glass slides. Heat-induced antigen retrieval was made with citrate buffer (pH 6.0) in a Pascal pressure chamber (125 °C/20 min). Endogenous peroxidase activity was blocked using a 10% hydrogen peroxide solution in methanol for 15 min. Primary antibodies were diluted in phosphate-buffered saline and incubated for 16 h (overnight) at 4 °C. The reaction was amplified using an anti-mouse/anti-rabbit detection system (Novolink Polymer Detection System, Leica Biosystems, Newcastle, UK) following the manufacturer’s instructions. Reagents were applied manually, and immunoreactivity was visualized with diaminobenzidine chromogen (DAB Substrate System, Dako, Carpinteria, CA, USA) for 3 min. Negative controls were obtained by substituting primary antibodies with normal serum. Breast tissue samples from women positive for ER, PR, and Ki67 and canine sweat gland carcinoma were used as positive controls (Nunes et al. 2021; Rollins-Raval et al. 2011; Sammarco et al. 2020).

**Table 1:** Immunohistochemical panel applied to noninvasive malignant mammary adenomyoepithelioma in a male cat

Antibody	Manufacturer	Clone	Dilution	Target cells
C-Kit	Dako	CD117	1:800	Epithelial (luminal)
Pan cytokeratin	Dako	AE1/AE3	1:500	Epithelial and myoepithelial
Cytokeratin 7	Dako	OV-TL 12/30	1:50	Epithelial (luminal)
GATA3	Cell Marque	L50-823	1:250	Epithelial (luminal)
P63	Dako	DAK-p63	1:100	Myoepithelial/basal
Estrogen receptor (ER)	Dako	1D5	1:50	Epithelial (luminal)
Progesterone receptor (PR)	Invitrogen	hPRa2	1:50	Epithelial (luminal)
Ki67	Dako	MIB-1	1:50	Proliferating epithelial and myoepithelial cells
Cox-2	Thermofisher	SP-21	1:50	Epithelial and myoepithelial

Quantitative analysis of immunostaining was performed in five HPF (40×). Positivity was identified by the presence of distinct brown staining in neoplastic cells. Staining was assessed as the percentage of positive cells, counting at least 500 cells in five HPF (Sammarco et al. 2020).

**Fig. 2:**
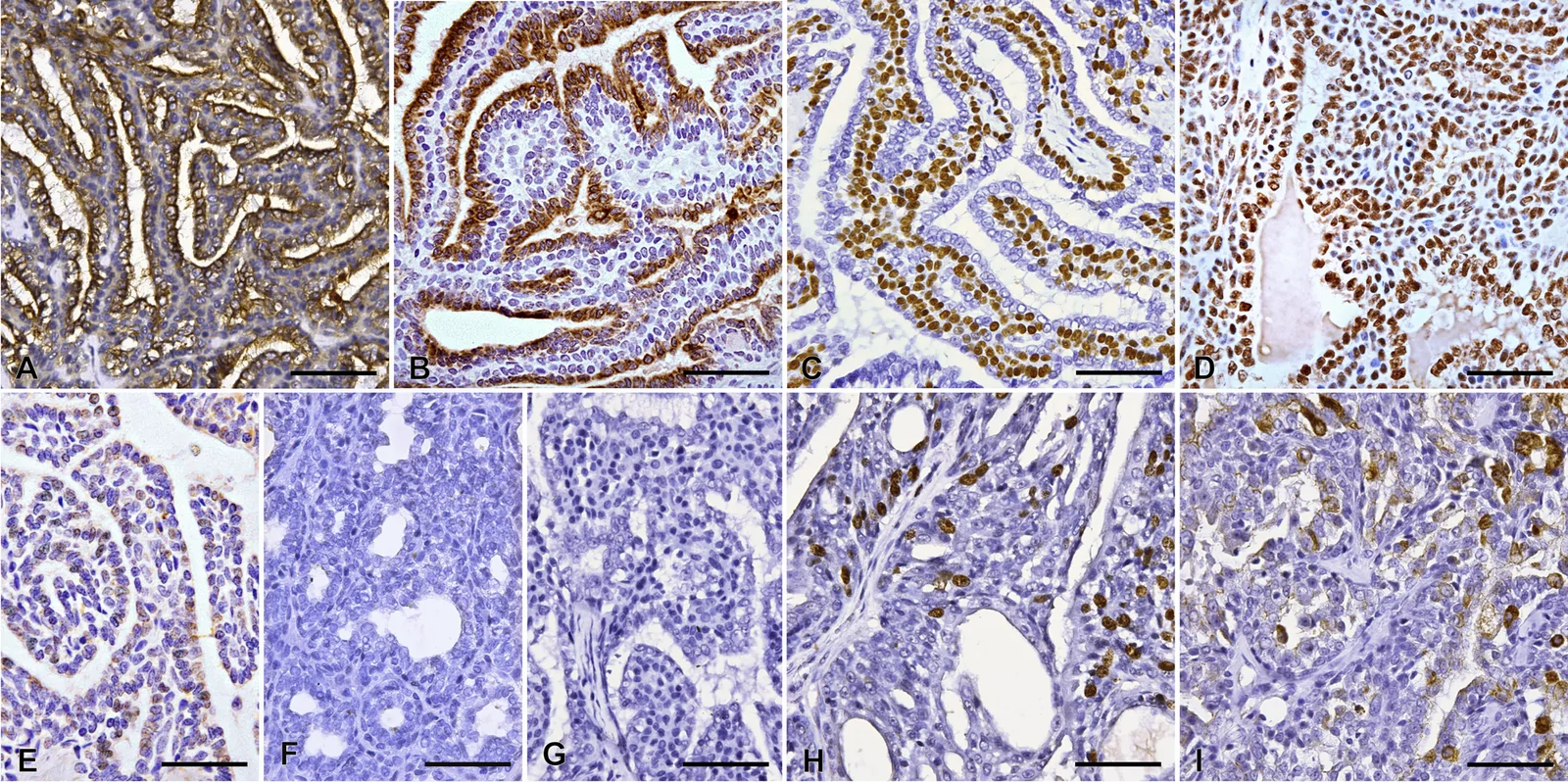
Immunohistochemical panel, noninvasive malignant mammary adenomyoepithelioma in a male cat. (**A**) Neoplastic epithelial cells with strong cytoplasmic labelling for CK AE1/AE3. DAB, Harris’ hematoxylin counterstain, bar: 50 μm. (**B**) Neoplastic epithelial cells with strong cytoplasmic labelling for CK7, Harris’ hematoxylin counterstain, bar: 50 μm. (**C**) Neoplastic myoepithelial cells with nuclear labelling for P63. DAB, Harris’ hematoxylin counterstain, bar: 50 μm. (**D**) Neoplastic epithelial cells with nuclear labelling for PR. DAB, Harris’ hematoxylin counterstain, bar: 50 μm. (**E**) Neoplastic cells with no labelling for GATA3. DAB, Harris’ hematoxylin counterstain, bar: 50 μm. (**F**) Neoplastic cells with no labelling for C-Kit. DAB, Harris’ hematoxylin counterstain, bar: 50 μm. (**G**) Neoplastic cells with no labelling for ER. DAB, Harris’ hematoxylin counterstain, bar: 50 μm. (**H**) Neoplastic epithelial and myoepithelial cells with nuclear labelling for Ki-67. DAB, Harris’ hematoxylin counterstain, bar: 50 μm. (**I**) Neoplastic epithelial and myoepithelial cells with cytoplasmic labelling for Cox-2. DAB, Harris’ hematoxylin counterstain, bar: 50 μm

Neoplastic epithelial cells showed diffuse cytoplasmic positivity for CK AE1/AE3 and CK7 (Fig. 2A, B). Over 90% of the neoplastic myoepithelial cells’ nuclei were positive for P63 (Fig. 2C), while approximately 90% of the epithelial cells were positive for PR (Fig. 2D). Epithelial and myoepithelial cells stained negative for GATA3, C-Kit and ER (Fig. 2E-G).

The Ki67 index was determined by counting the number of Ki67-positive cells per 500 neoplastic cells. Nuclear positivity for Ki-67 was observed in 20% of the neoplastic cells, both epithelial and myoepithelial (Fig. 2H).

For Cox-2, the analysis was performed according to the criteria established by Lavalle et al. (2009) for mammary carcinomas in female dogs. Positivity for Cox-2 was indicated by cytoplasmic staining, and the estimated percentage of positive cells was determined in five HPF (40×). The percentage of positive epithelial and myoepithelial cells was estimated according to the distribution and staining intensity to determine the total score by multiplying the distribution score by the intensity score. The total score ranged from 0 to 12; in the present case, the neoplasia presented a total score of 4, which was considered low (Fig. 2I).

Based on the macroscopic, microscopic, and immunohistochemical findings, the patient was diagnosed with noninvasive malignant mammary adenomyoepithelioma.

## Discussion

In the present case, the diagnosis of adenomyoepithelioma was based on the morphological characteristics of epithelial and myoepithelial cells in HE-stained sections, along with immunohistochemical marker expression, as described in human medicine (McLaren et al. 2005). Tumor cell positivity for cytokeratin’s and p63 confirmed the biphasic nature of the neoplasm, indicating epithelial and myoepithelial differentiation, respectively.

In adenomyoepitheliomas, the epithelial component forms nests or solid groups, tubules, trabeculae, and/or papillary projections (Goldschmidt et al. 2011; Jones et al. 2003; Nunes et al. 2021; Rakha et al. 2021). In the present case, the epithelial cells were arranged in a papillary pattern, with the myoepithelial component organized around them. Rakha et al. (2021) proposed a classification that recognizes malignant adenomyoepithelioma in situ (noninvasive) as a distinct category. These lesions retain the characteristic biphasic architecture of adenomyoepithelioma and exhibit malignant cytological and/or proliferative features while remaining confined to a noninvasive growth pattern. In the present case, the absence of invasion, necrosis, vascular invasion, and metastasis, together with the high mitotic index and elevated Ki-67 expression, supports classification within this spectrum.

The main histological criteria for adenomyoepithelioma malignancy include cellular and nuclear pleomorphism, high mitotic activity, and the presence of necrosis and invasion at the periphery of the tumor (Cassali et al. 2024). The tumor in the present case exhibited moderate pleomorphism, and no areas of invasion or necrosis were observed. Nevertheless, the mitotic index exceeded the limits established for classification as atypical adenomyoepithelioma in humans (5 mitoses per 10 HPFs) (Rakha et al. 2021), allowing it to be classified as a malignant neoplasm.

The classification of human adenomyoepitheliomas remains controversial, as these tumors encompass a broad morphological spectrum and exhibit variable biological behavior. Although the WHO Classification recognizes only benign and malignant forms of adenomyoepithelioma (WHO Classification of Tumours Editorial Board 2019), evidence indicates that some lesions classified as benign exhibit locally infiltrative growth, increased mitotic activity, and occasional cytologic atypia, without meeting all established histological criteria for malignancy (Tavassoli 1991; Hayes 2011). These observations support the concept that adenomyoepitheliomas present a wide biological spectrum (Rakha et al. 2021). In veterinary medicine, the classification of mammary adenomyoepitheliomas is also categorized only as “benign” and “malignant.” (Cassali et al. 2024). In this context, the absence of a formally recognized category for adenomyoepithelioma of low malignant potential represents a limitation of the current classifications. The tumor observed in the present case could be classified within this intriguing category, as it presents histopathological features suggestive of biological behavior more aggressive than expected for benign adenomyoepithelioma, despite not meeting all diagnostic criteria for malignant neoplasms.

The immunostaining index for Ki67 not only reflects cellular proliferative activity but can also serve as a prognostic biomarker in feline mammary carcinomas when its expression exceeds 14% (Cassali et al. 2024). The present tumor was considered noninvasive because it presented precise limits, was encapsulated, presented moderate pleomorphism, and did not present areas of necrosis, vascular invasion, or metastasis. However, the tumor showed a high mitotic index and elevated Ki-67 expression, indicating malignant cellular proliferation and correlating with a poor prognosis (Campos et al. 2015; Cassali et al. 2024).

Neoplastic cells were positive for the progesterone receptor and negative for the estrogen receptor. The association between hormonal disorders and breast tumors remains controversial. In male cats, some mammary tumors have been associated with the prior use of progestins, including medroxyprogesterone acetate (Jacobs et al. 2010; Pîrvu et al. 2024; Voorwald et al. 2021) and cyproterone acetate (Spugnini et al. 2010). Despite their controversial efficacy, these drugs are used for behavioral control (e.g., aggression, territorial marking), contraception, and certain skin diseases (feline eosinophilia and proliferative keratopathies) (Rodrigues et al. 2023). Mammary tumors diagnosed in cats with a history of prior progestin use include fibroepithelial hyperplasia, papillary adenoma and carcinomas (Jacobs et al. 2010; Pîrvu et al. 2024; Skorupski et al. 2005; Spugnini et al. 2010; Voorwald et al. 2021).

In male dogs, the association between Sertoli cell tumors and tubulopapillary mammary carcinomas has already been described (Machado et al. 2020). The cat in this study was neutered, and there was no information in its clinical history regarding the time of neutering or the presence of testicular lesions. Male dogs with benign mammary tumors, such as simple adenomas and fibroadenomas, present cells with intense PR-positive expression, and ER-negative expression (Mamom et al. 2012), similar to that observed in the cat in this report. The scarcity of mammary tumors in male animals makes it difficult to correlate the expression of hormone receptors with disease prognosis, as has already been established in females.

Immunohistochemical expression of Cox-2 has been considered a prognostic marker of mammary carcinomas in dogs and cats (Cassali et al. 2025; Lavalle et al. 2009; Millanta et al. 2006). Studies of adenomyoepitheliomas, particularly those affecting cats, are scarce. A report of six cases of malignant mammary adenomyoepithelioma in female cats demonstrated positivity for Cox-2 in two of the six cases (Nunes et al. 2021). In the present case, the tumor presented with low Cox-2 expression, demonstrating its favorable prognosis.

As the mammary gland is classified as an apocrine gland, neoplasms of other glands of the same type should be considered when making a differential diagnosis. In cats, carcinomas of sweat glands may exhibit a similar histological pattern to that of the mammary gland when arranged in solid, tubular, ductal or papillary patterns. Additionally, there may be proliferation of myoepithelial cells (Haziroglu et al. 2014). The micropapillary pattern has also been reported (Machida et al. 2011). However, immunohistochemical evaluation enables adequate differentiation between mammary and sweat gland neoplasms. In feline sweat gland carcinomas, expression of carcinoembryonic antigen (clone CD66 Ab-2) and of the cytokeratins AE1/AE2 in the epithelial cells and of vimentin in the myoepithelial cells has been described (Machida et al. 2011). Staining for carcinoembryonic antigen, S-100 and lecithin is considered decisive in diagnosing sweat gland neoplasms (Cocchia et al. 1981; Penneys et al. 1982; Tamaki et al. 1986). Studies on human neoplasms report that it is not possible to differentiate between breast and sweat gland neoplasms based solely on ER and PR immunostaining (Busam et al. 1999; Rollins-Raval et al. 2011; Wallace et al. 1995).

GATA3 and CK7 are useful complementary immunohistochemical markers in the differential diagnosis of epithelial neoplasms of mammary and sweat gland origin. In the present case, the neoplasm showed moderate to strong CK7 expression, a finding consistent with mammary origin, as sweat gland neoplasms are typically CK7-negative (Gloyeske et al. 2015). Conversely, GATA3 expression, which is usually preserved in sweat gland tumors, may be partially or completely lost in mammary neoplasms, particularly in estrogen receptor-negative tumors, as observed in this case (Asch-Kendrick and Cimino-Mathews 2016; Medeiros Souza et al. 2022).

Adenomyoepithelioma is a rare primary mammary tumor in cats, which may be benign or malignant and can exhibit varied morphological features. Accurate diagnosis requires a representative biopsy for histopathological and immunohistochemical evaluation. This case underscores the diagnostic challenges associated with atypical lesions and highlights the importance of recognizing histological subtypes of feline mammary neoplasms, especially in male cats.

## Data Availability

No datasets were generated or analysed during the current study.
